# Management of clinical stage T3/T4 bladder cancer: a review

**DOI:** 10.1007/s00345-025-05960-3

**Published:** 2025-09-29

**Authors:** Domenique Escobar, Mazyar Zahir, Chirag Doshi, Siamak Daneshmand

**Affiliations:** 1 Catherine and Joseph Aresty Department of Urology, Norris Comprehensive Cancer Center, University of Southern California, Los Angeles, CA USA; 2 USC Institute of Urology, 1441 Eastlake Ave, Suite 7416, Los Angeles, CA 90089 USA

**Keywords:** Locally advanced bladder cancer, Urothelial cancer, Urinary bladder neoplasms

## Abstract

**Introduction:**

Bladder cancer is a common malignancy in the United States and while the majority are non-muscle invasive at diagnosis, those with muscle-invasive and locally advanced disease can be challenging to manage. In addition, the prognosis is poorer in this group with high rates of recurrence following treatment. Clinical trials and advances in systemic therapy have helped to improve outcomes for these patients.

**Materials/methods:**

Articles were chosen for inclusion based on expert knowledge of the literature and PubMed literature searches for the relevant areas, with a focus on clinical trials. Appropriate articles were selected for inclusion by reviewing article titles, abstracts and full texts.

**Results:**

The standard of care for treatment of muscle invasive bladder cancer involves neoadjuvant chemotherapy followed by radical cystectomy. The NIAGARA trial recently changed the standard of care to include immunotherapy both in the neoadjuvant and adjuvant settings. Multiple clinical trials have assessed the potential benefit of adjuvant immunotherapy in patients with high-risk disease after radical cystectomy, leading to the approval of nivolumab in this setting. Improvements in staging and surveillance of these patients are necessary. The use of circulating tumor DNA and advances in imaging have also shown promise in prognostication and detection and monitoring of recurrence.

**Conclusions:**

Locally advanced bladder cancer is a challenging condition to manage, and while advances have been made in systemic therapy and biomarkers such as circulating tumor DNA, further investigation is needed to continue to improve outcomes for this group of patients.

## Introduction

Bladder cancer is a prevalent malignancy in the United States (US) with over 83,000 new cases diagnosed in 2024 [1]. While roughly 75% of new cases represent non-muscle invasive bladder cancer (NMIBC), the remainder are either muscle invasive (T2, MIBC) or metastatic at presentation [2]. Some patients with high risk NMIBC, particularly those with features such as HGT1 disease, variant histology, hydronephrosis and lymphovascular invasion, are at significant risk of progression, upstaging and occult lymph node metastases at radical cystectomy (RC) [3].

Management of MIBC can be challenging and prognosis is highly dependent on pathologic T and N stage [4]. Locally invasive disease, defined as tumors invading the perivesical soft tissue (T3), adjacent organs such as the prostate, uterus, vagina or the abdominal wall or pelvic side walls (T4), or lymph node positive disease (N+), have demonstrably poorer prognosis than those with organ confined (T2) disease, with post-cystectomy recurrence rates ranging from 20 to 30% in those with T2 disease to as high as 70% in those with N + disease [5, 6]. Determination of locally advanced disease is usually made with imaging, exam under anesthesia and pathology at transurethral resection (TUR). Cross-sectional imaging can identify perivesical involvement/inflammation, direct extension to other organs or lymphadenopathy. Exam under anesthesia involves a bimanual exam after TUR of the tumor and has been shown in various studies to offer important clinical staging information and concordance with pathologic stage [7, 8]. Pathology at TUR, particularly if prostatic urethral disease is present, also helps to delineate a patient’s stage. For example, prostatic ductal involvement would represent pTis disease, prostatic stromal involvement separate from the bladder tumor would represent pT2 disease and direct extension into the prostatic stroma from the bladder tumor would represent pT4 disease. These different disease states also portend different prognoses [9, 10].

The gold standard of treatment of MIBC (including T3/T4 disease) remains neoadjuvant chemotherapy (NAC) followed by RC. While recent advances in systemic therapy have shown promise, prognosis overall remains poor, calling for continued efforts to improve outcomes in these patients. In this review, we will provide a comprehensive overview of the management of T3 and T4 bladder cancer, including future directions.

## Materials/methods

A narrative (non-systematic) review was performed. Articles were chosen for inclusion based on expert knowledge of the literature and PubMed literature searches for the relevant areas, with a focus on recent clinical trials. Pertinent search terms included “locally advanced”, “bladder cancer”, “urothelial carcinoma”, “T3”, “T4”, “radical cystectomy”, “variant histology”, “neoadjuvant”, “adjuvant”, “chemotherapy”, including specific medication names such as “gemcitabine”, “cisplatin”, and “enfortumab”, “immunotherapy”, including specific medication names such as “atezolizumab”, “nivolumab”, and “pembrolizumab.” These terms were also searched in combination with terms related to the specific topics to follow. Specific clinical trial names were searched as well. Appropriate articles were selected for inclusion by reviewing article titles, abstracts and full texts.

## Results

### Neoadjuvant therapy

The recommendation for the use of NAC in MIBC is based on two large phase 3 randomized clinical trials that showed significant rates of pathologic downstaging and survival benefits of 5–6% for those who received NAC compared to those who did not [11–13]. In fact, those with T3/T4 disease appeared to benefit the most from NAC [11]. Both gemcitabine and cisplatin (gem/cis, generally 4 cycles) and dose-dense methotrexate, vinblastine, doxorubicin and cisplatin (ddMVAC, generally 6 cycles) have been studied and both are considered acceptable regimens. The SWOG1314 randomized trial that compared 4 cycles of ddMVAC with 4 cycles of gem/cis found no difference in pathologic complete response (pCR) or downstaging [14]. The prospective VESPER trial compared the two in the perioperative setting (both neoadjuvant and adjuvant) and included patients with pure urothelial carcinoma and either cT2-4aN0M0 disease (neoadjuvant group) or pTanyN1-2M0 disease (adjuvant group). The authors found no differences in 5-year overall survival (OS) in the cohort as a whole but did find improved 3-year progression-free survival (PFS) and 5-year OS rates in the neoadjuvant subgroup for those who received ddMVAC. However, ddMVAC does confer additional doses of platinum and potentially increased risks of adverse events [15, 16]. It is important to note that only about 16% of the cohort had T3 or T4 disease making it difficult to draw conclusions about which regimen is superior in this group. A large retrospective study looked specifically at this group and found a significantly higher rate of pCR with ddMVAC (28%) compared to gem/cis (14.6%) as well as higher OS with ddMVAC [17].

A patient’s candidacy for cisplatin-based NAC is generally dependent on their functional status, medical comorbidities and baseline renal function, with a glomerular filtration rate of 60 mL/min usually representing the lower limit for eligibility [18]. However, alternative thresholds are increasingly being used, particularly in larger trials. While it is estimated that ~ 60–70% of patients with MIBC are eligible for NAC, only about 20% receive it, for various reasons, including older age or comorbidities [19]. Thus, there is a need for additional therapies in the neoadjuvant setting and improvement in utilization for those who are candidates.

Enfortumab vedotin (EV) is an antibody-drug conjugate targeting Nectin-4, which is highly expressed in urothelial carcinoma. EV, either as single-agent therapy or in combination with the immunotherapeutic PD-1 inhibitor, pembrolizumab, has shown promise in phase 3 trials in patients with locally advanced or metastatic urothelial cancer with significant improvements in OS and PFS [20, 21].

These promising studies have since led to the investigation of these agents in the neoadjuvant setting. For example, the EV-103 phase 1b/2 clinic trial enrolled cisplatin-ineligible patients with cT2-T4aN0M0 disease, over 30% of whom had T3-T4 disease. Patients received neoadjuvant EV followed by RC. The pCR rate was 36.4%, the pathologic downstaging rate was 50% and the event-free survival (EFS) rates was 62% at 2 years [22]. The KEYNOTE-905/EV-303 study (NCT03924895) is a phase 3 trial assessing perioperative (“sandwich” treatment) pembrolizumab alone or in combination with EV in pts with MIBC (T2-T4aN0M0 or T1-T4aN1M0) who are ineligible for or decline cisplatin-based therapy. The primary endpoints of this trial are pCR and EFS [23]. The KEYNOTE-B15/EV-304 study (NCT04700124) is another phase 3 trial assessing combination EV/pembrolizumab (“sandwich” treatment) versus chemotherapy in cisplatin-eligible patients with MIBC (T2-T4aN0M0 or T1-T4aN1M0). The primary endpoints of this trial are also pCR and EFS [24]. These trials all included patients with ≥ 50% urothelial histology, thus allowing for some level of variant histology, an important point as those with variant histology have been historically excluded from trials in this arena.

The NIAGARA phase 3 randomized trial assigned patients to either neoadjuvant gem/cis plus durvalumab followed by RC and then adjuvant durvalumab (“sandwich” treatment) or gem/cis followed by RC. 60% in both arms were >T2N0, a unique strength of this study. Those who received the durvalumab combination therapy, regardless of baseline tumor stage, had significantly improved EFS and OS but with a stronger effect noted in the >T2N0 group [25]. These findings led to the very recent approval of this combination regimen for MIBC by the US Food and Drug Administration (FDA). This trial excluded those with pure variant or small-cell histology but allowed those with urothelial carcinoma with squamous or glandular differentiation. The latter group made up ~ 14–17% of the overall cohort and responded similarly to those with pure urothelial carcinoma.

Neoadjuvant therapy in variant histology can pose an additional challenge given the general underrepresentation of these histologies in many trials and lack of robust evidence. However, a recent review sought to organize the existing evidence in this space. For example, for pure squamous cell carcinoma, epirubicin or combination paclitaxel, carboplatin, and gemcitabine should be considered. In adenocarcinoma, NAC appears to confer favorable oncologic outcomes with some studies having assessed traditional urothelial carcinoma regimens and others 5-fluorouracil-based regimens. In sarcomatoid variants, upfront RC is recommended given the general lack of efficacy of systemic therapy [26]. The benefit of NAC in micropapillary disease remains controversial. One study found no difference in OS, RFS, or CSS in those who received cisplatin-based NAC compared to those who did not [27]. Other studies have shown modest response rates to immunotherapy or EV [28].

Other studies have assessed patients deemed to be unresectable with locally advanced or metastatic disease. The CheckMate 901 study was a phase 3 trial which assessed patients with previously untreated unresectable or metastatic urothelial carcinoma. Patients were randomized to receive either nivolumab plus gem/cis followed by nivolumab or gem/cis alone. Those in the nivolumab combination group experienced significantly better outcomes (OS, PFS and overall objective and complete response rates) [29]. The JAVELIN 100 trial was another randomized phase 3 clinical trial including patients with unresectable locally advanced or metastatic urothelial carcinoma who did not have disease progression with first-line chemotherapy. Patients were randomized to receive best supportive care with or without maintenance avelumab. OS and PFS were significantly improved in those who received maintenance avelumab [30]. While none of the patients in these studies went on to RC, these findings mark an important shift in the treatment paradigm of this patient population.

Despite these advances, management of locally advanced and/or clinically N + disease continues to pose a significant challenge for providers. While some may view these patients as metastatic and treat them with definitive systemic therapy, others may opt for neoadjuvant therapy followed by consolidative treatment (either RC or radiation). Typically, those with lymphadenopathy above the level of the pelvis will receive definitive systemic therapy but where exactly this anatomic line is drawn can be a gray area and up to the discretion of the treating provider. In addition, what determines whether a locally advanced bladder cancer is “resectable” is also often in the eye of the beholder and there may be variation across providers.

## Radical cystectomy

Radical cystectomy involves removal of the bladder, prostate and seminal vesicles in men and the bladder and possibly the adjacent reproductive organs (anterior vaginal wall, uterus, cervix, fallopian tubes and ovaries) in women. While there has been a shift towards reproductive organ-sparing procedures in select female patients given the benefits in sexual and urinary function in those with orthotopic diversion [31], this is not always feasible in locally advanced disease. However, one study found that patients with pathologic locally advanced disease (pT3, pT4 and N + at RC) who underwent reproductive organ-sparing surgery had similar oncologic outcomes (RFS, CSS, OS) to those who did not have organ-sparing procedures [32].

Another point of ongoing discourse is the comparison between open RC (ORC) and robotic-assisted RC (RARC). The robot-assisted radical cystectomy versus open radical cystectomy in patients with bladder cancer (RAZOR) trial found comparable 2-year PFS rates between the two approaches [33]. Another study utilizing the National Cancer Database found that that although mortality appeared higher in ORC compared to RARC, there were no statistically significant differences after adjusting for demographics and pathological tumor characteristics [34]. A large randomized clinical trial from the United Kingdom found no difference in oncologic outcomes between the two approaches but less thromboembolic and wound complications and improved number of days alive and out of the hospital in the RARC group [35].

The American Urological Association guidelines state that urethrectomy (immediate or delayed) should be performed in men if there is high grade cancer present at the urethral margin and in women if they are not undergoing orthotopic diversion to reduce the likelihood of a positive margin or recurrence [2]. However, this practice is variable, and the utility is not entirely clear. While some studies have found decreased local recurrence rates and improved survival in those who undergo prophylactic urethrectomy [36], others have not noted a survival benefit [37]. Interestingly, there is also evidence that urethral recurrence rates are lower in those with orthotopic neobladders compared to non-orthotopic diversions [38]. Thus, this practice is generally left to the discretion of the treating provider.

A pelvic LND is another critical facet of RC. This offers important staging information and confers a therapeutic benefit as well. At a minimum, the external and internal iliac and obturator lymph nodes should be removed and at least 10–15 lymph nodes should be removed for the LND to be considered sufficient [39]. A prospective, randomized trial assessed whether standard or extended LND (removal of deep obturator, common iliac, presacral, paracaval, interaortocaval, and para-aortic nodes) impacted outcomes in patients with T1 or T2-T4aM0 disease. Outcomes including RFS, CSS, and OS were not different between the two with some speculation that including those with T1 disease may have contributed to this finding. However, there were increased rates of lymphoceles in the extended group [40]. A subsequent larger prospective randomized trial, SWOG S1011, similarly sought to understand whether standard or extended LND improved OS and disease free-survival (DFS) in patients with localized MIBC (T2-T4a, N0-2). The authors found no difference between the two approaches in terms of oncologic outcomes but did identify higher complication rates in the extended LND group [41].

An area of ongoing research and controversy relates to whether the bladder can be preserved in patients with a clinical CR after neoadjuvant therapy. Several trials are underway to further explore this in combination with genetic testing and immunotherapy. However, existing evidence has shown unacceptably high rates of development of metastatic disease in those who underwent surveillance after a clinical CR and an increased mortality risk with delayed RC. Therefore, RC remains the standard of care [42].

## Chemoradiation

An acceptable alternative to RC for patients with localized MIBC is chemoradiation (chemoRT), also known as trimodal therapy. This involves maximal TUR of the bladder tumor with chemotherapy and external beam radiation therapy. Ideal candidates for chemoRT generally have localized disease, adequate bladder function, solitary tumors < 7 cm, no or only unilateral hydronephrosis, and minimal or no CIS [43]. While traditionally this option was reserved for patients medically unfit for or refusing RC, a recent, large, retrospective matched-cohort study of over 700 patients with cT2-T4N0M0 disease who were eligible for RC or chemoRT showed similar oncologic outcomes between the two groups [44]. While patients with T3-4 disease comprised a relatively small portion of the overall cohort in this study (and this group remains underrepresented in the literature in general), studies have aimed to assess how these patients fare with chemoRT. One study found that those who underwent chemoRT had significantly higher mortality rates at 5 years and increased risk of metastases compared to those who underwent RC[45]. However, another study that included 30 patients with T3-4 or N + disease who were not candidates for RC and were treated with systemic therapy (chemotherapy or immunotherapy) and definitive radiation found that this may be a reasonable alternative to RC. 40% of patients in this study had some component of variant histology. OS rates at 1- and 2-years were 73% and 61%, respectively; with better survival in the chemotherapy group compared to the immunotherapy group. 1-year PFS was similar between the two groups. Both hydronephrosis and variant histology were significant predictors of worse OS. 40% of the patients experienced some type of recurrence, the majority of which were distant metastases. 33% of the cohort was alive and disease-free at the last follow up [46]. Lastly, a large retrospective study assessed chemoRT in patients with variant histology (including 76% with squamous and/or glandular differentiation and 24% with other histologies) compared to pure urothelial carcinoma. The authors found no significant difference in CR, OS, CSS, or salvage cystectomy rates between the two groups. Of note, the presence of variant histology was not associated with CSS on multivariable analysis [47]. While there is a paucity of data in this space and additional research is needed, these results are encouraging for this group.

## Adjuvant therapy

Despite the progress made in the neoadjuvant space, some patients will still have aggressive pathology at RC and recurrence rates can be up to 50% within two years following RC [4, 48, 49]. There are also patients who cannot or do not receive any neoadjuvant therapy for a variety of reasons. Those with variant histology may be over-represented in these scenarios given propensity for upstaging, occult lymph node metastases and limited neoadjuvant therapy options [3, 26, 50], highlighting the need for advances in adjuvant therapies across the spectrum of MIBC.

While most patients who were not candidates for cisplatin-based chemotherapy pre-RC are not candidates post-RC, there is still a role for adjuvant chemotherapy in select patients. A recent systematic review and meta-analysis found that adjuvant cisplatin-based chemotherapy conferred a significant benefit in various oncologic outcomes including OS, RFS, and metastasis-free survival [51].

Adjuvant immunotherapy has also been an area of great research interest. For instance, the IMvigor010 trial was a randomized phase 3 trial assessing adjuvant atezolizumab versus observation in patients with high-risk disease at RC: ypT2-4a or ypN + disease if the patient had received NAC or pT3-4a or pN + disease if the patient had not received NAC. There was no difference in the primary endpoint of DFS between the two groups, but this was the first completed trial which explored the role of immunotherapy in the adjuvant setting [48]. The CheckMate 274 randomized phase 3 trial assessed the use of adjuvant nivolumab versus placebo in the same patient population and found significantly improved DFS in the nivolumab group [52], noted in both the intention-to-treat population and among patients with a PD-L1 expression level of 1% or more. This led to its approval by the US FDA in August 2021 for patients regardless of PD-L1 status, but in Europe it is only approved for those with PD-L1 expression levels of 1% or more.

Lastly, the AMBASSADOR randomized phase 3 trial assessed the use of adjuvant pembrolizumab in the same patient population (although this trial also allowed variant histology and microscopically positive surgical margins). The authors found significantly improved DFS (but not OS on preliminary analysis) in the pembrolizumab group (regardless of PD-L1 status), although the frequency of adverse events with this medication was significantly higher than that with nivolumab in the CheckMate 274 trial [53].

**Fig. 1:**
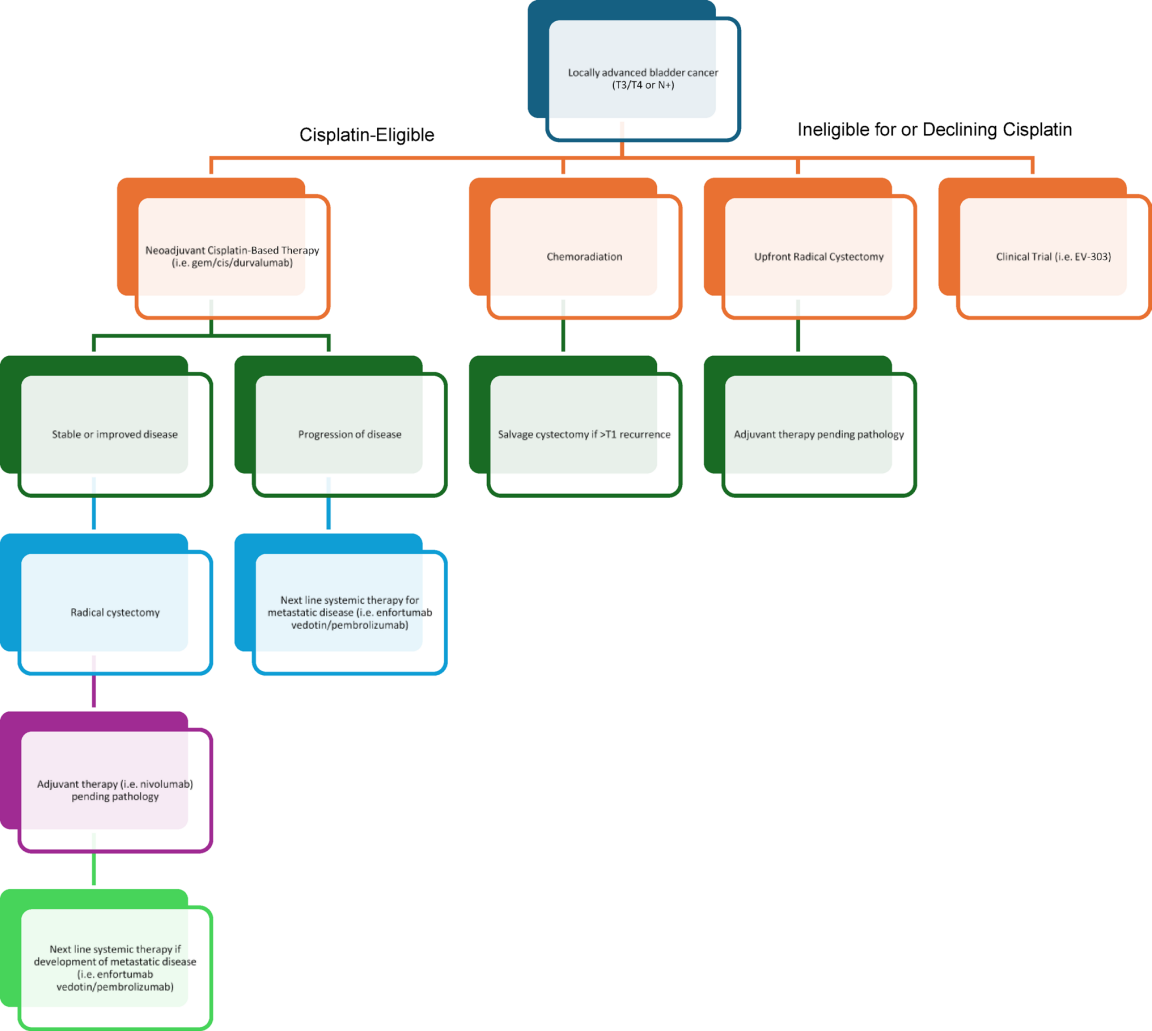
Treatment algorithm for locally advanced urothelial bladder cancer

The noteworthy clinical trials in the neoadjuvant, surgical and adjuvant settings are summarized in Table 1. A generalized algorithm for the treatment of patients with locally advanced disease is shown in Fig. 1.

### Future directions

Advances in other areas, such as biomarkers, have great potential to change and improve how these patients are managed. Circulating tumor DNA (ctDNA), which has been used in various tumor types, has shown significant promise in bladder cancer. Dead tumor cells release DNA into the bloodstream which can be detected and utilized as a biomarker. ctDNA has been shown to be associated with disease burden and can predict progression and recurrence, often before traditional methods (i.e., imaging and routine lab work) detect recurrence. A recent meta-analysis highlighted the strengths of ctDNA in patients with MIBC. For example, ctDNA-positive state was significantly associated with poorer OS (HR 4.51), PFS (HR 4.5), and RFS (HR 6.56). In addition, clearance of ctDNA after treatment was a predictor of very favorable RFS (HR 0.24) [54]. In fact, although the previously discussed IMvigor010 trial was a negative trial, exploratory data showed that atezolizumab reduced the risk of death by roughly 40% in patients who were ctDNA positive after RC. This prompted the development of the prospective IMvigor011 trial to validate these findings. IMvigor011 is a phase 3, randomized, double-blinded study to evaluate the efficacy and safety of adjuvant atezolizumab compared with placebo in patients with MIBC who are ctDNA positive and are at high risk of recurrence following RC[55]. Thus, this test has the potential to help understand the prognosis on a more detailed level and to help target the “window” of minimal residual disease.

Another point to consider in future clinical trials is the potential role of Nectin-4 as a surrogate marker for treatment response to EV. A recent study on tissue specimens from patients with metastatic urothelial carcinoma revealed that 26% of the cohort had Nectin-4 amplifications. Notably, 96% of these patients showed an objective response to EV (82% partial and 14% complete), compared to only 32% (29% partial and 3% complete) in patients without Nectin-4 amplification [56].

Improved imaging modalities are also needed as conventional imaging can be imperfect for staging, particularly in identifying N + disease. Previous studies have reported that up to 25% of patients with pathologic N + disease did not have abnormal lymphadenopathy on pre-RC imaging [57]. Conventional fluorine-18 2-fluoro-2-deoxy-D-glucose (^18^F-FDG) positron emission tomography (PET) imaging has been shown to be better at detecting metastatic lesions compared to conventional imaging [58] and other radiotracers have also been evaluated. For example, a study assessing an early experience with ^68^Ga-FAP-2286 PET, a novel fibroblast activation protein (FAP) binder, found that it was highly sensitive in patients with localized and metastatic disease. It was also found to be effective in identifying metastatic lesions across a variety of anatomic sites, including subcentimeter lymph nodes that would not have raised suspicion on conventional imaging. In 3 patients, ^68^Ga-FAP-2286 PET helped to identify false-positive findings on conventional FDG PET and false-negative findings on conventional cross-sectional imaging [59]. Magnetic resonance imaging (MRI) has also shown promise in bladder cancer, although more so in helping to distinguish non-muscle invasive from muscle-invasive tumors [60]. The VI-RADS system has been developed for standardized reporting of MRI findings in bladder cancer [61].

## Discussion and conclusions

Overall, locally advanced bladder cancer can be challenging to accurately stage and manage, with high rates of recurrence and overall poor prognosis. The gold standard remains NAC followed by RC, however multiple ongoing clinical trials are evaluating the utility of immunotherapy or other chemotherapy agents in the neoadjuvant setting. Some patients will also require adjuvant therapy, either with chemotherapy or immunotherapy and multiple trials have been completed in this space. In addition, several trials are also investigating combinations of treatments such as the addition of immunotherapy to chemoRT [62] and bladder preservation with immuno-RT in those with MIBC and a clinical CR after neoadjuvant therapy (SWOG 2427/BRIGHT trial). However, because accurate staging remains difficult in these patients (both initially and after neoadjuvant therapy), bladder preservation in those with a clinical CR after neoadjuvant therapy is not currently recommended due to high rates of metastases and increased mortality risk in those delaying RC [42].

Several innovations allowing for more personalized approaches to the management of these patients, including biomarkers such as ctDNA and advances in imaging, have been developed with the goal of improving staging accuracy and offering important prognostic information. Similarly, genome sequencing in variant histology may also help to guide treatment decisions in this understudied group, especially as targeted therapies continue to be developed [63]. However, it is important to note their limitations including potential discordance in tumor molecular profiles between the primary site and metastases [64].

In conclusion, significant work remains to improve oncologic outcomes in patients with locally advanced bladder cancer and this will likely include personalized approaches including novel biomarkers and clinical trials.

**Table 1:** Pivotal clinical trials in clinical stage T3/T4 bladder cancer

Trial name, publication year	Trial ID	Design	Population/eligibility	Intervention	Primary endpoint	Key results
*Neoadjuvant therapy*						
Javelin 100, 2024	NCT02603432	Phase 3	Unresectable or metastatic urothelial carcinoma	Neoadjuvant GC without vs. with adjuvant avelumab	OS PFS (Median 19 months follow-up)	OS time: ***sig*** A/GC: 21.4 months GC: 14.3 months PFS time: ***sig*** A/GC: 3.7 months GC: 2.0 months
Niagara, 2024	NCT03732677	Phase 3	cT2-T4aN0-1M0	Perioperative (“Sandwich”) durvalumab plus GC vs. GC alone	EFS	2-yr EFS: ***sig*** D/GC: 67.8% GC: 59.8% 2-yr OS: ***sig*** D/GC: 82.2% GC: 75.2%
Keynote-869/EV-103, 2024	NCT03288545	Phase 1b/2	cT2-T4aN0M0	Neoadjuvant EV	pCR	pCR: 36.4% pDS: 50% 2-yr EFS: 62%
Vesper, 2021, 2024	NCT01812369	Phase 3	cT2-4aN0M0 (neoadjuvant) and pTanyN1-2M0 (adjuvant)	ddMVAC vs. GC perioperative chemotherapy	3-yr PFS	3-yr PFS in whole cohort: *not sig* dd-MVAC (64%) GC (56%) 5-yr OS in NAC: ***sig*** dd-MVAC (66%) GC (57%) 3-yr PFS in NAC: ***sig*** dd-MVAC (66%) GC (56%)
Checkmate 901, 2023	NCT03036098	Phase 3	Unresectable or metastatic urothelial carcinoma	Neoadjuvant GC without vs. with adjuvant nivolumab	OS PFS (Median 33.6 months follow-up)	OS time: ***sig*** N/GC: 21.7 months GC: 18.9 months PFS time: ***sig*** N/GC: 7.9 months GC: 7.6 months
SWOG1314, 2021	NCT02177695	Phase 2	cT2-T4a N0 M0	ddMVAC vs. GC neoadjuvant chemotherapy	Predictive value of COXEN score for pCR or pDS	pCR: *not sig* ddMVAC (28%) GC (30%) pDS: *not sig* ddMVAC (47%) GC (40%)
*Radical cystectomy*						
SWOG S1011, 2024	NCT01224665	Phase 3	cT2-T4N0-2M0	Standard vs. Extend LND	DFS OS	5-yr DFS: *not sig* Extended: 56% Standard: 60% 5-yr OS: *not sig* Extended: 59% Standard: 63%
Extended vs. limited lnd in bc patients undergoing RC, 2019	NCT01215071	Phase 3 (superiority design ~ 15%)	HGT1 or cT2-T4aM0	Limited vs. Extend LND	RFS CSS OS	5-yr RFS: *not sig* Extended: 65% Limited: 59% 5-yr CSS: *not sig* Extended: 76% Limited: 65% 5-yr OS: *not sig* Extended: 59% Limited: 50%
RAZOR, 2018	NCT01157676	Phase 3 (non-inferiority design ~ 15%)	cT1-T4N0-1M0 or refractory CIS	Robot-assisted vs. open radical cystectomy	PFS	2-yr EFS: ***sig*** RARC: 72.3% ORC: 71.6%
Trial to compare robotically assisted radical cystectomy with open radical cystectomy, 2022	NCT03049410	Phase 3	Non-metastatic, node status ≤ N1, undergoing radical cystectomy	Robot-assisted with intracorporeal diversion vs. open radical cystectomy	Number of days alive and out of the hospital within 90 days of surgery	Number of days: ***sig*** RARC: 82 days ORC: 80 days Note: no differences in RFS or OS between approaches
*Adjuvant Therapy*						
Ambassador, 2024	NCT03244384	Phase 3	>ypT2/ypN+/margin + or >pT3/pN+/margin +	Adjuvant pembrolizumab vs. observation	DFS OS (Median 44.8 months follow-up)	DFS time: ***sig*** Pembrolizumab: 29.6 months Observation: 14.2 months
Checkmate 274, 2021	NCT02632409	Phase 3	ypT2-4a/ypN+ or pT3-4a/pN+	Adjuvant nivolumab vs. placebo	DFS (Median 20.9 months follow-up)	DFS time: ***sig*** Nivolumab: 20.8 months Placebo: 10.8 months
IMvigor010, 2020	NCT02450331	Phase 3	ypT2-4a/ypN+ or pT3-4a/pN+	Adjuvant Atezolizumab vs. observation	DFS (Median 21.9 months follow-up)	DFS time: *not sig* Atezolizumab: 19.4 months Observation: 16.6 months
*Trials in progress*						
Keynote-905/EV-303	NCT03924895	Phase 3	cT2-T4aN0M0 or cT1-T4aN1M0	Perioperative (“Sandwich”) pembrolizumab vs. EVP	EFS	-
Keynote-B15/EV-304	NCT04700124	Phase 3	cT2-T4aN0M0 or cT1-T4aN1M0	EVP vs. cisplatin -based neoadjuvant chemotherapy	EFS	-
IMvigor011	NCT04660344	Phase 3	(y)pT2-4aN0M0 or (y)pT0-4aN + M0	Adjuvant atezolizumab vs. placebo in ctDNA + patients	DFS	-^**†**^
SWOG 2427/BRIGHT	NCT07061964	Phase 2	cT0-1 without multifocal CIS following NAC	RT + pembrolizumab	BI-EFS	-

^**†**^ Primary unpublished results from IMvigor011 have shown a statistically significant and clinically meaningful increase in DFS and OS for ctDNA-positive patients treated with atezolizumab[65].

*A/GC*: Avelumab/Gemcitabine/Cisplatin, *CSS*: Cancer Specific Survival, *ctDNA*: circulating tumor DNA, *DFS*: Disease Free Survival, *D/GC*: Durvalumab/Gemcitabine/Cisplatin, *ddMVAC*: dose-dense methotrexate/vinblastine/doxorubicin/cisplatin, *EFS*: Event Free Survival, *EV*: Enfortumab vedotin, *EVP*: Enfortumab vedotin/Pembrolizumab/*GC*: Gemcitabine/Cisplatin, *NAC*: Neoadjuvant Chemotherapy, *N/GC*: Nivolumab/Gemcitabine/Cisplatin, *ORC*: Open Radical Cystectomy, *OS*: Overall Survival, *pCR*: pathologic complete response, *pDS*: pathologic downstaging, *PFS*: Progression Free Survival, *RARC*: Robot-assisted Radical Cystectomy, *RFS*: Recurrence Free Surivival, *RT*: Radiotherapy.

## Data Availability

No datasets were generated or analysed during the current study.
